# The Exercise–Affect–Adherence Pathway: An Evolutionary Perspective

**DOI:** 10.3389/fpsyg.2016.01285

**Published:** 2016-08-25

**Authors:** Harold H. Lee, Jessica A. Emerson, David M. Williams

**Affiliations:** Department of Behavioral and Social Sciences, Brown University School of Public HealthProvidence, RI, USA

**Keywords:** exercise adherence, affect, evolution, perceived utility, hedonic valence, ultimate causes

## Abstract

The low rates of regular exercise and overall physical activity (PA) in the general population represent a significant public health challenge. Previous research suggests that, for many people, exercise leads to a negative affective response and, in turn, reduced likelihood of future exercise. The purpose of this paper is to examine this exercise–affect–adherence relationship from an evolutionary perspective. Specifically, we argue that low rates of physical exercise in the general population are a function of the evolved human tendency to avoid unnecessary physical exertion. This innate tendency evolved because it allowed our evolutionary ancestors to conserve energy for physical activities that had immediate adaptive utility such as pursuing prey, escaping predators, and engaging in social and reproductive behaviors. The commonly observed negative affective response to exercise is an evolved proximate psychological mechanism through which humans avoid unnecessary energy expenditure. The fact that the human tendencies toward negative affective response to and avoidance of unnecessary physical activities are innate does not mean that they are unchangeable. Indeed, it is only because of human-engineered changes in our environmental conditions (i.e., it is no longer necessary for us to work for our food) that our predisposition to avoid unnecessary physical exertion has become a liability. Thus, it is well within our capabilities to reengineer our environments to once again make PA necessary or, at least, to serve an immediate functional purpose. We propose a two-pronged approach to PA promotion based on this evolutionary functional perspective: first, to promote exercise and other physical activities that are perceived to have an immediate purpose, and second, to instill greater perceived purpose for a wider range of physical activities. We posit that these strategies are more likely to result in more positive (or less negative) affective responses to exercise, better adherence to exercise programs, and higher rates of overall PA.

## Introduction

Regular physical activity (PA) improves physical health (Haskell et al., 2007; Donnelly et al., 2009) and mental health (Schuch et al., 2011), and prolongs life span (Reimers et al., 2012). However, only 51.6% of US adults meet the national guideline of 1000 kcal/week of PA (Centers for Disease Control and Prevention [CDC], 2013) and approximately 5.3 million people die globally each year due to lack of PA (Lee et al., 2012). Given its well-known benefits, increasing rates of regular PA remains an important public health challenge.

Until recently, research on PA promotion has tended to emphasize cognitive determinants of PA adherence, such as expected health outcomes, self-efficacy, behavioral intentions, and social norms (Marcus et al., 2006; Williams and Marcus, 2012). However, in the past 10–20 years there has been greater attention to affective processes among PA promotion researchers (Ekkekakis, 2003; Ekkekakis et al., 2005; Kiviniemi et al., 2007; Rhodes et al., 2007, 2009; Williams et al., 2008, 2012; Schneider et al., 2009; Kwan and Bryan, 2010a,b; Conner et al., 2011; Rose and Parfitt, 2012; Baldwin et al., 2013). Much of this research has focused on the way people feel in response to intentional exercise—a form of PA performed for the purposes of health and fitness.

Despite the ubiquitous media message that “exercise feels good,” it is now clear that the relationship between exercise and affect is not so simple (Emerson and Williams, 2015). While most people tend to *feel good after exercise*, many people—including a large proportion of inactive and unfit individuals who are the targets of PA promotion efforts—*feel bad during exercise* (Ekkekakis et al., 2011). Thus, it is more accurate to say that while almost everyone likes to be finished with exercise, many people actually dislike exercising.

In addition to these scientific findings, evidence that many people dislike physical exertion can be seen by the high rate of escalator or elevator use in lieu of the stairs, the use of human conveyor belts at airports, and the tendency for people to circle around parking lots trying to find a close place to park. Other evidence comes from the wide use of effort-saving devices such as remote controls, electric garage door openers, and electric can openers. It is hard to argue that these automated products are simply used to save time, or that they are only used to assist those who would otherwise be physically incapable of manually performing the relevant tasks.

Perhaps not surprisingly, research has also shown that, consistent with ancient and contemporary theories of human behavior (Hobbes, 1651/2008; Bentham, 1789/2007; Mill, 1861/2012; Usener, 1887/2010; Young, 1952; Cabanac, 1971; Kahneman et al., 1997), those who experience a more negative affective response to exercise are less likely to repeat it in the future, and thus more likely to drop out of exercise promotion programs (Rhodes and Kates, 2015). Thus, from a public health perspective, it is critically important to understand why many people have a negative affective response to a behavior—physical exercise—that is good for their health. That is, why do many people dislike exercising?

The aim of this paper is to attempt to answer this question by taking an evolutionary perspective. We will argue that the negative affective response to exercise is a manifestation of an evolved tendency to avoid energy expenditure that served no immediate adaptive function. In modern environments, this evolved psychological mechanism has led to difficulty with exercise adherence and overall low rates of PA.

In making the above arguments we first highlight the distinction between proximate and ultimate (i.e., evolutionary) causes of behavior. We then apply this distinction in an attempt to understand the evolutionary basis for the low rates of compliance with exercise programs and corresponding low rates of PA. Specifically, we posit that negative affective response to exercise is the proximate psychological mechanism through which the ultimate cause of preventing unnecessary energy expenditure influences poor compliance with exercise programs. We then consider the characteristics of exercise—its intensity and lack of perceived utility—that contribute to a negative affective response to exercise among many individuals. In this context we discuss the concept of *perceived utility of PA* as a cognitive mechanism that evolved to signal when PA had an immediate adaptive payoff, such as when chasing prey, fleeing from predators, or engaging in social play. Finally, we discuss the implications of this model for designing exercise promotion interventions.

## Proximate Versus Ultimate Causes of Behavior

The biologist Ernst Mayr distinguished between proximate and ultimate causes of behavior (Mayr, 1963; for a similar formulation, see Tinbergen, 1963; for a recent discussion of Mayr’s distinction in the context of psychological science, see Scott-Phillips et al., 2011). When trying to understand the causes of behavior, behavioral scientists are often concerned with proximate causes: *How* does the behavior occur? Proximate causes of behavior refer to the *biological or psychological mechanisms* that control the behavior in the here and now. The vast majority of behavioral science research addresses questions of proximate causation, which involve elucidation of physiological, psychological, or social phenomena that explain the target behavior. For example, elucidating the causal mechanisms of unhealthy eating may involve research on individual variability in metabolic processes, food preferences and cravings, ubiquity of environmental food cues, and social norms related to eating (e.g., Roth et al., 2001; Blundell et al., 2005; Davis et al., 2007).

However, according to Mayer, to gain a full understanding of the causes of behavior, it is also necessary to consider ultimate causes: *Why* does the behavior occur? Ultimate causes of behavior refer to the *functional significance* of the behavior from an evolutionary perspective. Questions of ultimate causation assume that biological evolution is largely driven by the process of natural selection whereby genetically based traits that increase the odds of survival and reproduction are more likely to be perpetuated in future generations. Such questions are the subject matter for the fields of sociobiology (Wilson, 2000), behavioral ecology (Cronk, 1991), and evolutionary psychology (Cosmides and Tooby, 1987; Plotkin, 1997).

Importantly, in exploring questions of ultimate causation, one should distinguish between the functional significance of the behavior in the here-and-now versus its functional significance at the time that the pattern of behavior evolved. This point is easily illustrated when considering the caloric overconsumption that is largely responsible for the recent (on an evolutionary timescale) obesity epidemic. Human tendency to consume high fat and sugary foods when available (e.g., Power and Schulkin, 2013; Speakman, 2016) increased the odds of survival and reproduction among human evolutionary ancestors, because such foods were scarce and in those ancestral environments provided a dense energy source (e.g., Keskitalo et al., 2007). However, the same tendencies are maladaptive in modern environments where such foods are abundant (though only “maladaptive” in an evolutionary sense to the extent that such patterns of eating reduce fecundity).

The answers to questions about proximate and ultimate causes of behavior can be integrated. Proximate causes of behavior are the biological and psychological mechanisms through which the ultimate causes of behavior have their influence on the behavior. That is, for an adaptive pattern of behavior to evolve there must be a biological and/or psychological trait(s) that provides the mechanism through which the organism executes the adaptive behavior. The biological/psychological mechanism(s) answers the proximate *how* question. To continue with the example from above, the fact that energy-dense (i.e., sweet and fatty) foods taste good to most humans and thus motivate us to consume such foods is the proximate biological/psychological mechanism through which the evolutionarily adaptive (at the time that it evolved) tendency to eat high fat and sugary foods operates.

## Proximate Causes of Low Rates of Exercise Adherence

To date, most research investigating the behavior of physical exercise has examined questions of proximal causation: How does exercise behavior occur, or not occur? That is, what causes people to engage in exercise, or not, in the here and now?

As noted in the introduction, much of this research has focused on cognitive factors, such as expected outcomes of exercise, self-efficacy, social norms, and behavioral intentions. These cognitive factors help to explain how people successfully set goals, formulate intentions, make plans, and overcome barriers to exercise. But why is it so difficult to exercise to begin with? People do not need to set goals, formulate intentions, make plans, and work hard to overcome obstacles to watch television on a regular basis. Most of us regularly end up in front of the television on most days without any of this cognitive effort (Ekkekakis et al., 2016). So why is it so easy to watch television but so hard to exercise?

The answer, as we have argued above, is that many people generally dislike exercising. That is, we humans tend to have a negative affective response during many types of intentional physical exercise. And the fact that many people dislike exercising is a main reason for the low rates of exercise participation and overall PA. Thus, the human tendency to have a negative affective response to most types of exercise is a proximate cause of the low rates of exercise and PA in the general population.

## Ultimate Causes of Low Rates of Exercise Adherence

The tendency for people to engage in, or avoid, physical exercise can also be examined from an evolutionary perspective by addressing the question of ultimate causation: Why does exercise behavior occur, or not occur? That is, what is the functional significance of avoiding exercise behavior?

Certainly, it is not functionally adaptive to avoid exercise in today’s modern environment. In fact, exactly the opposite is true. Regular exercise has numerous health benefits that prolong life and increase fertility. However, to address the question of functional significance, it is necessary to conceptualize “function” with respect to the prevailing conditions under which the target behavior evolved. Our evolutionary ancestors—whether protohumans or earlier ancestors—had to perform vast amounts of PA just to obtain food and avoid predators. Thus, if anything, our ancestors, along with other animal species, had the opposite energy balance problem from the one faced by modern humans: taking in enough energy to maintain energy balance given the energy expenditure necessary for survival and reproduction.

Expending energy through extraneous PA that had no purpose other than to expend energy (i.e., exercise) would have decreased the survival and reproductive fitness of human evolutionary ancestors. As a result, selection pressures would have favored the genetic predisposition to conserve energy by avoiding PA that did not serve a direct adaptive function, such as obtaining food, fleeing from predators, or engaging in important social interactions.

In sum, the functional significance of the human tendency to avoid intentional physical exercise is that it allowed us to conserve energy, thus leading to decreased risk of potential energy deficits, and increased likelihood of survival and reproduction among our evolutionary ancestors (for a more detailed argument along these lines, see Lieberman, 2015).

## Negative Affective Response to Exercise as an Adaptive Psychological Mechanism

We have thus far argued that the ultimate cause of the low rates of physical exercise is the human tendency to conserve energy; and the proximate cause of the low rates of physical exercise is the human tendency to respond to physical exercise with negative affect. An integration of these causes suggests that the human tendency to respond to physical exercise with negative affect is the proximate psychological mechanism through which the ultimate cause of conserving energy has its effects on the behavior of avoiding physical exercise.

**FIGURE 1:**
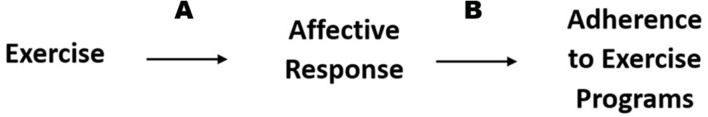
**The exercise–affect–adherence pathway**. Path **(A)** refers to the domain-specific influence of exercise behavior on affective response. Path **(B)** refers to the domain-general influence of affective response on future exercise behavior.

This genetically endowed *exercise–affect–adherence pathway* (Williams, 2008) can be broken down into two dyadic relationships (**Figure 1**): the exercise–affect relationship (path A) and the affect–adherence relationship (path B). In the following sections, we will first more precisely define affective response to exercise. Then, we will discuss, in turn, the affect–adherence and exercise–affect relationships.

## Defining Affective Response to Exercise

According to the circumplex model, *core affect* can be characterized along two dimensions: valence (i.e., good versus bad) and arousal (i.e., high versus low; Russell, 1980; for a discussion of the “positive affect” and “negative affect” dimensions of the “rotated circumplex model” and the confusion caused by these labels, see Ekkekakis, 2013). Distinct affective states, such as specific moods (e.g., peaceful, depressed, irritable, energized) and emotions (e.g., anxious, joyful, angry, sad) represent some combination of core affective valence and arousal that can be arranged in four quadrants. For example, anxiety represents “bad” valence and “high” arousal, whereas joy represents “good” valence and “high” arousal. In addition to core affect, moods and emotions may also involve a triggering environmental stimulus, cognitive appraisals of such stimuli, specific patterns of physiological responses, and facial expressions.

When considering affective response to exercise as a potential determinant of future exercise behavior, rather than as an outcome in its own right (e.g., effects of exercise training on depressive symptoms), it is useful to focus on core affective valence (i.e., feeling good versus bad). The reason affective valence is useful for this purpose is because according to numerous ancient and contemporary theories of *psychological hedonism* (Hobbes, 1651/2008; Bentham, 1789/2007; Mill, 1861/2012; Usener, 1887/2010; Young, 1952; Cabanac, 1971; Kahneman et al., 1997; see also Affective Response to Exercise as a Determinant of Exercise Adherence) people are more likely to repeat behaviors that make them feel good and avoid behaviors that make them feel bad. Thus, when examining how people respond to exercise, the critical issue is whether they feel good or bad rather than whether they feel, for example, anxious versus embarrassed or joyful versus excited. Accordingly, when discussing affective response to exercise we will focus on *core affective valence*.

Another important aspect of our conceptualization of affective response to exercise has to do with timing. Studies assessing the way people feel before and after exercise generally support the conclusion that acute bouts of exercise improve affective states (Yeung, 1996). These findings appear to create a paradox as such positive affective responses to exercise should lead to high rates of exercise participation and adherence (Wininger, 2007). Yet, as we have discussed above, rates of regular exercise and overall PA are dismal (Tucker et al., 2011).

Hall et al. (2002) have pointed out that this apparent paradox can be explained by the fact that assessments of affect are often administered prior to and following, but not during the exercise task (for a review, see Ekkekakis and Petruzzello, 1999). According to learning theory, immediate consequences of behavior are more predictive of future behavior than delayed consequences (Neef et al., 1994). The subjective affective response experienced during exercise is more immediate than feelings experienced after the exercise has been completed, which may also include the affective response to completing exercise (Hall et al., 2002). Thus, when discussing affective response to exercise we will focus on core affective valence experienced *during* exercise rather than after exercise.

Finally, when considering affective *response* to exercise, it is critical to consider not just how people feel during exercise, but how their affect *changes* from *before* exercise to *during* exercise.

Following from the above arguments, we conceptualize affective response to exercise as *the shift in core affective valence from pre-exercise to during-exercise*.

## Affective Response to Exercise as a Determinant of Exercise Adherence

The affect–adherence aspect of the posited exercise–affect–adherence pathway (**Figure 1**, path B) is consistent with psychological hedonism (aka the hedonic principle): people tend to pursue behaviors that lead to pleasure and avoid behaviors that lead to displeasure or pain. This basic principle of human behavior has been observed since the ancient Greeks (Usener, 1887/2010) and has been restated in the writings of Hobbes (1651/2008), Bentham (1789/2007), and Mill (1861/2012) and more recently in work by Young (1952), Cabanac (1992), and Kahneman et al. (1997) among others.

There is empirical evidence supporting the principle of psychological hedonism in the context of the exercise domain—i.e., the affect–adherence link. Recent studies have shown that affective responses recorded *during* individual exercise sessions at baseline and 6 months of an exercise promotion program were predictive of adherence to the exercise program 6 and 12 months later, even when controlling for ratings of perceived exertion (Williams et al., 2008, 2012). A recent review of studies showed similar findings, also highlighting that affective response *during* rather than immediately following exercise was predictive of future exercise behavior (Rhodes and Kates, 2015).

In addition to the handful of studies supporting the affect–adherence link in the context of exercise behavior, there is considerable evidence for the broader principle of psychological hedonism in research on other health-related behaviors, particularly addiction and eating behavior (Williams and Evans, 2014). Thus, the affect–adherence link (**Figure 1**, path B) in the posited exercise–affect–adherence pathway is not specific to the behavioral domain of exercise (i.e., domain-specific), but is instead consistent with the general principle of psychological hedonism that operates across behavioral domains. That is, the principle of psychological hedonism is *domain-general*.

As a domain-general principle, psychological hedonism—i.e., the tendency to pursue behaviors that lead to pleasure and avoid behaviors that lead to pain—has its own set of proximate and ultimate causes that go beyond the domain of exercise and thus beyond the scope of this paper. Numerous previous authors have written about the evolutionary underpinnings of psychological hedonism, as well as its proximate psychological and neurobiological mechanisms (e.g., Broom, 2001; Lemos, 2004; Panksepp, 2010). For an integration of proximate and ultimate causes of psychological hedonism in the context of health-related behavior, see Williams and Ruse (forthcoming).

## Affective Response to Exercise: The Importance of Intensity and Perceived Utility

We have thus far characterized affective response to exercise as “often negative” and claimed that many people dislike most types of exercise. Of course, the exercise–affect association is more nuanced than that. It is certainly true that many people dislike most types of physical exercise. But, *most* of us like at least *some* types of exercise. So what are the factors that cause someone to have a positive, negative, or indifferent affective response to exercise? And what is the potential functional significance of such affective responses? The answers to these questions are critical given the principle of psychological hedonism discussed above, the low rates of physical exercise in the general population, and the health consequences of an inactive lifestyle.

Just as an evolutionary approach was used to address the broader question of why many people avoid physical exercise, such an approach can be used to understand affective response to different types of exercise. To take this evolutionary approach it is necessary to consider what might be the functional significance of positive versus negative affective responses to different types of exercise, as well as the characteristics of different physical activities that would have made them adaptive to engage in during human evolutionary development.

**FIGURE 2:**
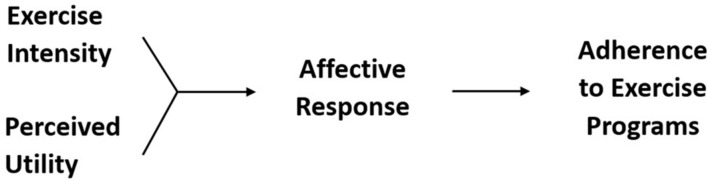
**Affective response as a function of exercise intensity and perceived utility**.

Taking such an evolutionary perspective, we argue that the two factors that are most relevant to affective response to exercise are the intensity of the exercise and its perceived utility (**Figure 2**). This follows from our arguments that the evolved tendency to avoid physical exercise has to do with the functional significance of reducing *unnecessary* energy expenditure. Energy expenditure is, of course, a function of the intensity and duration of PA. And, the *necessity* of exercise, we will argue, is a function of its perceived immediate utility. Thus, we consider the negative affective response to *exercise* to be a manifestation of the more general tendency toward a negative affective response to *PA that is perceived to be unnecessary or of low utility*.

### Affective Response to Exercise of Different Intensities

From a functional perspective, intensity of exercise is critical because it determines energy expenditure over a fixed duration. In most classification systems, intensity of exercise refers to workload or energy expenditure per unit time, or physiological stress, such as percent of maximal heart rate or maximal oxygen consumption. However, Ekkekakis and colleagues (Ekkekakis, 2003; Ekkekakis et al., 2011) have argued that when examining affective response to exercise the most useful way to characterize exercise intensity is with respect to the ventilatory threshold (VT). The VT is the point at which the body transitions from anaerobic to aerobic metabolism and can be, albeit crudely, ascertained via the talk test: if you cannot carry on a conversation while exercising then you have exceeded the VT. More sophisticated assessment of the VT involves examination of expired air to determine the ratio of ventilation to oxygen consumption, which will begin to increase exponentially at the VT (Posner et al., 1987). Alternatively, blood lactate levels can be obtained to determine the lactate threshold, which generally coincides with the VT.

The reason that percentage of VT is a useful indicator of intensity when studying affective response to exercise is because the VT represents a transition point beyond which exercise cannot be maintained without disruption of homeostasis and severe bodily harm. That is, humans cannot engage in an all-out sprint for more than 100–200 m.

Using percent of VT as the indicator for exercise intensity, a pattern of findings is emerging in which:

(a) Exercise below the VT (e.g., slow walking) tends to result in a positive affective response,
(b) Exercise approaching but not exceeding the VT (e.g., brisk walking or, for some people, jogging) results in an affective response that varies from person to person and from one situation to the next, and
(c) Exercise exceeding the VT (e.g., jogging or, for some people, fast running or sprinting) that is continued for more than 10–20 s results in a nearly universal negative affective response (Ekkekakis et al., 2011).

According to the dual-mode model, there are two distinct psychobiological pathways—interoceptive and cognitive—that influence affective response to exercise (Ekkekakis, 2003; Ekkekakis and Acevedo, 2006). At exercise intensities above the VT, affective response is dependent on interoceptive cues that accompany the transition from aerobic to anaerobic metabolism. This includes feelings of fatigue and discomfort from accumulation of lactate and hydrogen ions as they dissociate from lactic acid, accelerated breakdown of creatine phosphate (McCann et al., 1995), the inhibition of glycolysis and glycogenolysis (Spriet et al., 1989), and the interference with the calcium triggering of muscle contractions (Favero et al., 1995). Because the physiological consequences of prolonged exercise exceeding the VT are universal among humans, the accompanying feelings of pain and displeasure in response to exercise of an intensity that exceeds the VT are also nearly universal (with the possible exception of individuals with congenital insensitivity to pain). This may be considered a specific case of the domain-general human tendency to experience pain resulting from tissue damage.

Thus, the functional significance of affective response to exercise exceeding the VT is relatively straight-forward and is explicitly stated in the context of the dual-mode model (Ekkekakis, 2009a,b). The negative affective response serves the function of providing a signal to decrease intensity or discontinue the exercise before serious bodily damage occurs (for a review, see Noakes, 2012).

But what can account for the variability in affective response to exercise approaching the VT? According to the dual-mode model, interoceptive factors play a lesser role in determining affective response to exercise that does not exceed the VT. Instead, when exercise intensity is approaching or below the VT then cognitive factors, such as perceived consequences of exercise, social norms, etc., play a larger role in determining affective response—i.e., the second “mode” in the dual mode model. Because there is greater variability among humans in these cognitive factors, the affective response to exercise that approaches but does not exceed the VT shows greater variability from person to person and in different situations.

The scant available research on cognitive determinants of affective response to exercise has thus far shown that social environment and self-efficacy are predictive of affective response to exercise of intensities approaching or below the VT (McAuley et al., 2000). These cognitive factors are important to understanding why some people may have a negative affective response to moderate intensity exercise, with implications for exercise promotion interventions. However, we propose that greater understanding of the variability in affective response to exercise that does not exceed the VT can be achieved by taking an evolutionary perspective. Specifically, we posit perceived utility as an important cognitive mechanism that determines affective response to exercise (**Figure 2**).

### Perceived Utility as a Determinant of Affective Response to Exercise

What determines affective response to exercise that is below or approaching the VT? This question has immense public health implications. If we can understand when and why people have a negative affective response to exercise, then we can develop interventions to promote more positive affective responses to exercise and thus make exercise adherence more likely.

One of the problems with intentional exercise as a form of PA is that it often has no immediate purpose other than to expend energy. Although energy expenditure, for its own sake, has positive long-term health consequences in most modern environments, it was not adaptive throughout the vast majority of evolutionary history stretching back to human hunter/gatherers, protohumans, and beyond (Gluckman and Hanson, 2008). Although some types of exercise, such as sports, may serve an additional purpose, many types of exercise, such as running on a treadmill or walking around a track, exemplify PA that would have been unnecessary and thus maladaptive in ancestral times (Lieberman, 2015). Moreover, the health benefits of PA in modern environments are indirect and temporally distal.

The perception that physical exertion serves some *immediate* purpose may be a key factor in determining affective response to exercise. Indeed, earlier we argued that energy expenditure was maladaptive *except when it was necessary to obtain food, avoid predators, engage in reproductive behavior, or engage in other important social activities.* Thus, an important cognitive factor that may influence affective response to physical exertion is the *perceived immediate utility of PA*.

Perceived utility may operate through both reflective and automatic pathways consistent with dual-processing models (Evans, 2008; Williams and Evans, 2014). The reflective sense of perceived utility comes from conscious consideration of the immediate outcome or goal of the PA. Thus, physical activities that have a clear and immediate purpose or goal, such as walking or biking for transportation, may result in a more positive affective response than walking or biking for no immediate purpose (**Figure 2**).

The automatic sense of purpose may result in performance of physical activities that mimic behaviors that would have served an immediate adaptive purpose among our evolutionary ancestors, even if such behaviors have no functional purpose in the here-and-now. For example, many leisure sports (e.g., rugby, soccer, basketball) and play behaviors (e.g., tag) involve chasing a ball or chasing or fleeing from an opponent, which mimic the chasing and fleeing behaviors necessary for survival in ancestral environments. Such physical activities may be more likely to result in positive affective responses than physical activities that lack these qualities (Jackson and Csikszentmihalyi, 1999; Chen et al., 2001). This automatic sense of purpose and its effects on action is consistent with the affordance-competition model in which action-selection is integrated with, rather than preceded by, conscious decision-making (Cisek, 2007; Cisek and Kalaska, 2010; Smits et al., 2014).

### Perceived Utility, Exercise Intensity, and Affective Response to Exercise

Perceived utility may have a dimensional quality that interacts with exercise intensity to influence affective response to exercise (**Figure 2**).

We hypothesize that when perceived utility is at the extreme low end, affective response to exercise will be negative regardless of exercise intensity. The available evidence suggests that humans tend to have a positive affective response to exercise that is below the VT. However, from an evolutionary perspective, prolonged exercise—even if well below the VT—still results in energy expenditure and thus may still have been maladaptive among human evolutionary ancestors particularly if it had no adaptive function and was continued for a significant duration. It follows that a negative affective response even to light intensity physical exertion may still occur when the exertion serves no other purpose. For example, to get up and change the channel on the television, take the stairs, or park far away when there is no reason not to simply use a remote control, take the elevator, or find a close parking spot. That is, people may universally respond positively to light intensity exercise as long as they perceive that it has some immediate utility.

When perceived utility is at the extreme high end, affective response may be positive—at least for brief periods—even when exercise intensity exceeds the VT. For example, while maximal exertion typically results in a negative affective response, such negative responses may be blunted if someone is sprinting to get on a bus that is about to leave. Likewise, affective response to maximal exertion is likely to be more positive in the context of team sports or running competitions.

In the middle range of the dimension, perceived utility may be enough to result in a positive affective response depending on the intensity of the exercise. Thus, at very low intensities even a meager perception of utility may lead to a positive affective response. For example, it is possible that complying with a researcher’s request provides enough of a purpose for engaging in light intensity exercise to turn what might otherwise be a negative affective response to a light intensity exercise into a positive affective response. However, affective response to exercise at higher intensities is likely to be negative if perceived utility is modest.

This conceptualization of the interaction between perceived utility and PA intensity is consistent with the dual-mode model in that perceived utility is a cognitive factor that plays a larger role when exercise intensity does not exceed the VT.

## Implications

One of the main points of the present paper is that the human tendency to have a negative affective response to PA that has no immediate utility—including many types of intentional exercise—is an innate tendency that is likely universal among humans. Likewise, the tendency to avoid PA that results in a negative affective response is consistent with the domain-general innate human tendency to pursue behaviors that lead to pleasure and avoid behaviors that lead to pain or discomfort.

The fact that these tendencies—which together represent the exercise–affect–adherence pathway—are innate does not mean that they are unchangeable. Indeed, humans can make conscious decisions about their behavior and thus are capable of overcoming even the strongest innate behavioral tendencies. Nonetheless, strong innate behavioral tendencies are *difficult* to overcome. Thus, it may be difficult to attempt to change the human tendency to dislike PA that has no immediate utility or the human tendency to avoid PA that has previously resulted in a negative affective response.

Instead, we propose an approach based on an evolutionary functional perspective: to (a) promote exercise and other physical activities that are perceived to have an immediate purpose, or (b) attempt to instill greater perceived purpose for a wider range of physical activities. We posit that these strategies are more likely to result in more positive (or at least less negative) affective responses to exercise, better adherence to exercise programs, and higher rates of overall PA.

One general strategy for improving adherence to exercise programs is to promote physical activities that are already perceived to have an immediate purpose, as these activities are more likely to result in a positive affective response and thus improved adherence. This may include increasing activities such as walking or cycling for transportation, or relying on gardening for producing fresh fruits and vegetables. Other examples may include promoting activities that mimic the once necessary activities of hunting (chasing) prey and escaping (fleeing) predators. For example, many leisure sports (e.g., rugby, soccer, basketball) and play behaviors (e.g., tag) involve chasing and/or fleeing and chasing and fleeing leisure activities are observed in both Eastern and Western history as well as in the present-day (Mechikoff and Estes, 2006). Other examples include interactive video games like Wi, Pokemon Go, etc. We hypothesize that these activities will result in a more positive affective response than activities of the same intensity that do not have the fleeing, chasing, or social bonding characteristics.

A second general strategy is to modify activities so that they have an immediate purpose. Perhaps the most relevant application of this idea is the use of financial incentives to increase exercise behavior. Consistent with behavioral economics theory (Kahneman, 2003), use of financial incentives provides an immediate purpose for physical exercise when there is no other functional purpose and given that the health benefits of exercise are uncertain and distal. Theoretically based concerns that financial incentives may undermine intrinsic motivation (Deci et al., 1999; Frey and Jegen, 2001) have largely been dispelled by consistent data showing that in most situations financial incentives complement rather than undermine intrinsic motivation (Cerasoli et al., 2014; Shaw and Gupta, 2015). Moreover, the undermining of intrinsic motivation is irrelevant for most health-related behaviors, including exercise, for which there is low baseline intrinsic motivation (Promberger and Marteau, 2013). Indeed, a recent systematic review shows preliminary support for the use of monetary incentives to increase exercise behavior (Strohacker et al., 2014).

## Limitations

### Is This a “Just-so Story”?

Although an evolutionary perspective may open up new avenues to thinking about the exercise–affect–adherence relationship, an inherent weakness is that hypotheses regarding functional significance are difficult to test empirically. It is impossible to recreate the ancestral environments in which the exercise–affect–adherence relationship evolved. While it *is* possible to generate hypotheses that are logically *consistent with* what we currently know about the exercise–affect–adherence relationship, as well as educated guesses about the nature of ancestral environments, a *post hoc* explanation that fits the existing data falls well short of an *a priori* hypothesis in terms of scientific viability.

It is for this reason that *post hoc* hypotheses about the evolution of modern traits are often referred to by skeptics as “just-so stories” (Gould and Lewontin, 1979; Kipling, 2013). For example, we may conjecture *post hoc* that the giraffe has a long neck because those giraffes born with long necks were more likely to reach the top-most leaves on trees and thus more likely to survive in times of drought and pass the long-neck genetic predisposition to their offspring. However, other explanations also fit the data and thus are just as viable. Perhaps giraffes evolved long necks because it allowed them to spot predators from far away, or long necks could have evolved as a weapon in intraspecies sparring matches, or as a sexual selection characteristic. The sparse available evidence points to the first of these alternative explanations (Cameron and du Toit, 2007), but is hardly conclusive.

Similarly, we may generate alternative explanations for the evolution of the exercise–affect–adherence relationship. For example, a viable alternative explanation for the negative affective response to exercise that approaches the VT—instead of positing an energy conserving function—is that such a response is due to the effects of evaluative conditioning. That is, exercise that approaches the VT often immediately precedes exercise that exceeds the VT, which, for reasons discussed above, automatically results in a negative affective response, presumably because of the immediate dangers of acute overexertion. Thus, exercise that approaches the VT (the conditioned stimulus) may come to elicit a negative affective response (the conditioned response) because it has become associated with exercise that exceeds the VT (the unconditioned stimulus), which reliably leads to a negative affective response (the unconditioned response). Though speculative, our point here is simply to illustrate how one can easily generate *post hoc* alternative hypotheses.

Given this, how can we produce evidence in support of our evolutionarily informed hypothesis regarding the functional significance of the negative affective response to moderate intensity exercise? Multiple authors have devised schemes for overcoming this problem (Cosmides and Tooby, 1987; Laland and Brown, 2011). Most important is that a posteriori evolutionary hypotheses (or theories) must be useful for generating *a priori* hypotheses and, when relevant, guiding practical interventions.

Our a posteriori hypothesis about the functional significance of a negative affective response to exercise and the role of perceived utility, like all scientific hypotheses, cannot be proven beyond all doubt. However, the evolutionary perspective we have outlined herein is useful for formulating *a priori* hypotheses about what sort of physical activities will result in more positive affective responses as well as what intervention strategies are likely to lead to improved adherence to exercise promotion programs. To the extent that these hypotheses are supported the overarching a posteriori evolutionary hypothesis also gains support.

### Dangers of a Misunderstood Evolutionary Approach

Applications of Darwinian evolutionary ideas can lead to defiance and outrage if misinterpreted. Thus, let us be clear. We are *not* suggesting that because most (if not all) humans have a genetic predisposition toward negative affective response to unnecessary PA that we are *predestined* to be physically inactive. Human (and all other animal) behavior is a function of the *interaction* between genetic predispositions *and* environmental conditions. Indeed, this is a main point of our thesis: it is only because of human-engineered changes in our environmental conditions (i.e., it is no longer necessary for us to work for our food) that our predisposition to avoid unnecessary physical exertion has become a liability. Thus, it is well within our capabilities to reengineer our environments to once again make PA necessary or, at least, to serve an immediate functional purpose.

Likewise, we are *not* suggesting that the tendency toward negative affective response to exercise is the *only* reason for the low rates of PA in the general population. There are other reasons why some people do not engage in regular exercise. For example, lack of access to safe and affordable places to exercise is a common barrier (Walsh et al., 1999), and some people anticipate feeling embarrassed or lack the social support to engage in physical activities (Anderson et al., 2006; Grieser et al., 2006). These alternative reasons for the low rates of exercise complement rather than compete with our evolutionary hypothesis.

## Conclusion

Despite our unique ability among animal species to significantly modify our environment, we humans are still members of the animal kingdom. We share with all animal species the genetically engrained tendency to be efficient when it comes to expending energy.

We hypothesized herein that negative affective response to exercise, particularly exercise that approaches or exceeds the VT, is an adaptive psychological mechanism that evolved because—in combination with the domain general tendency to avoid displeasure and pain—it minimized unnecessary PA among our evolutionary ancestors. Nonetheless, our ancestors needed to act when there was an immediate and compelling purpose, such as when obtaining food, fleeing from predators, or engaging in social bonding and reproduction. Thus, we further hypothesized that affective response to exercise, and other forms of PA, will be more positive when the perceived immediate utility of the PA is high.

In today’s environment, the human tendency to have a negative affective response to unnecessary physical exertion is directly responsible for the difficulty adhering to exercise programs and the corresponding low rates of PA in the general population. Although we no longer live in conditions that require us to expend energy to achieve basic survival needs, we can create conditions under which physical exertion is perceived to have an immediate purpose. This can be accomplished either by promoting activities that have an inherent utility (e.g., walking for transportation) or that mimic activities that served a purpose in ancestral environments (e.g., sports and games that involve chasing and fleeing), or by artificially providing an immediate utility for exercise (e.g., via monetary incentives).

## Author Contributions

HL conceived of the hypothesis with input from DW and JE. HL and JE wrote an early draft of the manuscript. DW revised and completed the writing of the manuscript. All authors read and approved the final manuscript.

## Conflict of Interest Statement

The authors declare that the research was conducted in the absence of any commercial or financial relationships that could be construed as a potential conflict of interest.
